# Phylogenetic and Evolutionary Analyses of the Frizzled Gene Family in Common Carp (*Cyprinus carpio*) Provide Insights into Gene Expansion from Whole-Genome Duplications

**DOI:** 10.1371/journal.pone.0144037

**Published:** 2015-12-16

**Authors:** Chuanju Dong, Likun Jiang, Wenzhu Peng, Jian Xu, Shahid Mahboob, Khalid A. Al-Ghanim, Xiaowen Sun, Peng Xu

**Affiliations:** 1 CAFS Key Laboratory of Aquatic Genomics and Beijing Key Laboratory of Fishery Biotechnology, Centre for Applied Aquatic Genomics, Chinese Academy of Fishery Sciences, Beijing, China; 2 College of Fisheries and Life Science, Shanghai Ocean University, Shanghai, China; 3 Department of Zoology, College of Science, King Saud University, Riyadh, Saudi Arabia; 4 Department of Zoology, GC University, Faisalabad, Pakistan; Institute of Hydrobiology, Chinese Academy of Sciences, CHINA

## Abstract

In humans, the frizzled (FZD) gene family encodes 10 homologous proteins that commonly localize to the plasma membrane. Besides being associated with three main signaling pathways for cell development, most FZDs have different physiological effects and are major determinants in the development process of vertebrates and. Here, we identified and annotated the FZD genes in the whole-genome of common carp (*Cyprinus carpio*), a teleost fish, and determined their phylogenetic relationships to FZDs in other vertebrates. Our analyses revealed extensive gene duplications in the common carp that have led to the 26 FZD genes that we detected in the common carp genome. All 26 FZD genes were assigned orthology to the 10 FZD genes of on-land vertebrates, with none of genes being specific to the fish lineage. We postulated that the expansion of the FZD gene family in common carp was the result of an additional whole genome duplication event and that the FZD gene family in other teleosts has been lost in their evolution history with the reason that the functions of genes are redundant and conservation. Through the expression profiling of FZD genes in common carp, we speculate that the ancestral gene was likely capable of performing all functions and was expressed broadly, while some descendant duplicate genes only performed partial functions and were specifically expressed at certain stages of development.

## Introduction

The frizzled (FZD) genes were first identified genetically in *Drosophila melanogaster*, where mutations of FZDs were reported to disrupt the polarity of epidermal cells and cause a planar cell polarity phenotype in the adult fly [1]. FZDs have been identified in primitive metazoans, including the sponge *Suberites domuncula* [2], and in *Hydra vulgaris* [3], but have not been described in protozoans. FZD genes have been shown to encode receptors for the Wnt proteins that define a large family of extracellular signaling molecules found throughout the animal kingdom [4–6] and FZDs regulate fundamental aspects of development in vertebrates.

Three main signaling pathways for cell development are activated by agonist-activated FZD proteins: the Wnt/β-catenin pathway; the Wnt/calcium pathway; and the planar cell polarity pathway [7]. The canonical Wnt/β-catenin pathway is characterized by stabilization of β-catenin protein in response to ligand binding; the Wnt/calcium pathway is defined by the ability of overexpressed Wnts and FZDs to cause increases in intracellular calcium; and the planar cell polarity pathway is defined by the set of genes that, when mutated, result in defects in the polarity of cells in a planar tissue [1].

FZD proteins are found exclusively at the plasma membrane. They are located at the surface of Wnt-responsive cells, although recent evidence has suggested that they may be internalized as part of a mechanism for regulating the extracellular level of Wnt protein and/or the cellular response to Wnts [8, 9]. The tissue-specific expression of FZD genes is complex, given that numerous FZDs have been described in metazoans. In general, FZD genes are widely and dynamically expressed and, indeed, it is rare to find a cell that does not express one or more FZD. Specific expression patterns of FZDs have been described in various model organisms [10, 11]. FZDs are major determinants in the development process of vertebrates and most FZDs have different physiological effects. In humans, the FZD gene family encodes 10 FZD proteins, all of which have distinct molecular functions. Many studies have characterized the functions of FZDs, especially in human and several other model species. For example, *FZD1* and/or *FZD2* mutations were found to cause defects in neural tube closure and disorientation of inner ear sensory hair cells, which indicated that FZD signaling was involved in diverse tissue closure processes, and these defects account for some of the most common congenital anomalies in human [12]. *FZD3* was reported to control axon guidance in the brain and spinal cord [13–15]. *FZD5* was shown to be required for yolk sac, placental angiogenesis, and survival of thalamic neurons in the parafascicular nucleus [16, 17]. *FZD6* was reported to control the orientation of hair follicles together with *FZD3* [18]. *FZD7* was found to be up-regulated in the gastric cancer cell line MKN7 and in a case of primary gastric cancer [19]. *FZD4* and *FZD8* were shown to control ureteric growth in the developing kidney [20], and both *FZD4* and *FZD9* were linked to modulate follicular development and maturation in the developing mammalian ovary [21–24].

In contrast to the 10 FZD genes present in the human and mouse genomes, there are much fewer FZD genes in invertebrates; for example, in *Caenorhabditis* there are three and in *Drosophila* there are four FZD genes [25], suggesting a significant expansion of the FZD gene family in vertebrates. The duplicated FZD genes have broad expression profiles in various tissues and cell types with dynamic combinations that facilitate the complex development processes in vertebrates. Compared with tetrapods, all the teleost fishes experienced a third round of whole genome duplication (3RWGD), also referred to as teleost-specific genome duplication, which generated the complex genomes found in teleosts. In addition to the teleost-specific 3RWGD event, a fourth round of whole genome duplication (4RWGD) occurred in some teleost species, Salmonidae (salmonids and trouts) [26, 27] and Cyprinidae (bony fishes including common carp) [28, 29], which added to the complexity of the genome organization in these species. The completion of the whole genome sequences of many teleosts allowed us to assess the complexity and organization of the FZD genes in teleosts. In particular, the reference genome of common carp, the first allotetraploid genome, has been published recently [30], providing the potential to compare FZD genes between tetraploid common carp and diploid teleosts, such as medaka and zebrafish, as well as tetrapods.

Common carp is one of the most widely cultured freshwater fish species in the world [31, 32]. Aquaculture production of common carp has increased parallel to the increase of global aquaculture production of freshwater fishes. Because of its importance in aquaculture and biology, various genomic and genetic studies of common carp have been performed in the past decades and abundant genome resources and data have been collected, including a large number of expressed sequence tags [33], bacterial artificial chromosome end sequences [34], comprehensive transcriptome sequences [35, 36], single nucleotide polymorphisms [37], and genetic and physical maps [38, 39], as well as the completely sequenced and annotated genome [30]. The aim of this study was to perform phylogenetic, evolutionary, and expression analyses of the FZD gene family to verify the whole genome sequence assembly and annotation [40], as well as to illustrate gene fates after the recent 4RWGD event in common carp. Our study has provided insights into gene duplication, divergence post multiple rounds of WGDs, and gene expression profiles in different tissues and stages in common carp.

## Result and Discussion

### FZD gene prediction and characterization

A total of the 26 FZD genes were identified in the recently published reference genome of common carp and various transcriptome data [30, 41–43]. The 26 genes were distributed on 11 chromosomes and eight scaffolds (Table 1) in the common carp genome and revealed substantial gene family expansion compared with other vertebrates. For instance, there are 10 FZD genes in the human, mouse, and clawed frog genomes and from nine to 14 FZD genes in other sequenced teleost genomes such as nine in spotted gar, 10 each in medaka and coelacanth, 11 in stickleback, and 14 in zebrafish.

**Table 1:** Nomenclatures and structures of FZD genes from common carp, zebrafish and human genomes.

	Common carp FZD	No. of exons in common carp	No. of exons in zebrafish	No. of exons in human
1	FZD1a	1	2	1
2	FZD1b	2	-	-
3	FZD2a	2	1	1
4	FZD2b	2	-	-
5	FZD3a	6	7	7
6	FZD3b	5	7	-
7	FZD4a	6	2	2
8	FZD4b	2	-	-
9	FZD4c	1	-	-
10	FZD5a	1	1	2
11	FZD5b	1	-	-
12	FZD6	1	2	7
13	FZD7a	3	1	1
14	FZD7b	1	2	-
15	FZD7c	2	-	-
16	FZD7d	2	-	-
17	FZD8a	1	1	1
18	FZD8b	3	1	-
19	FZD8c	1	-	-
20	FZD8d	1	-	-
21	FZD9a	2	1	1
22	FZD9b	1	1	-
23	FZD9c	1	-	-
24	FZD9d	1	-	-
25	FZD10a	2	1	1
26	FZD10b	1	-	-

Thirteen of the 26 FZD genes of common carp retained a single exon, eight retained two exons, and five retained three or more exons (S1 Fig). In the zebrafish gene, eight FZD genes had a single exon, four had two exons, and two had seven exons, while, in the human genome, six had single exon, two had two exons, and two had three exons or more (Table 1).

### Phylogenetic analysis and nomenclature of FZD gene family in common carp

In the evolution of higher eukaryotes, WGDs followed by polyploidization, as well as gene loss, have been an important recurrent process. Ancient WGDs, inferred from analyzed sequenced genomes and comparative genomics, are prevalent and recurring throughout the evolutionary history of higher eukaryotic lineages [44].

**Fig 1:**
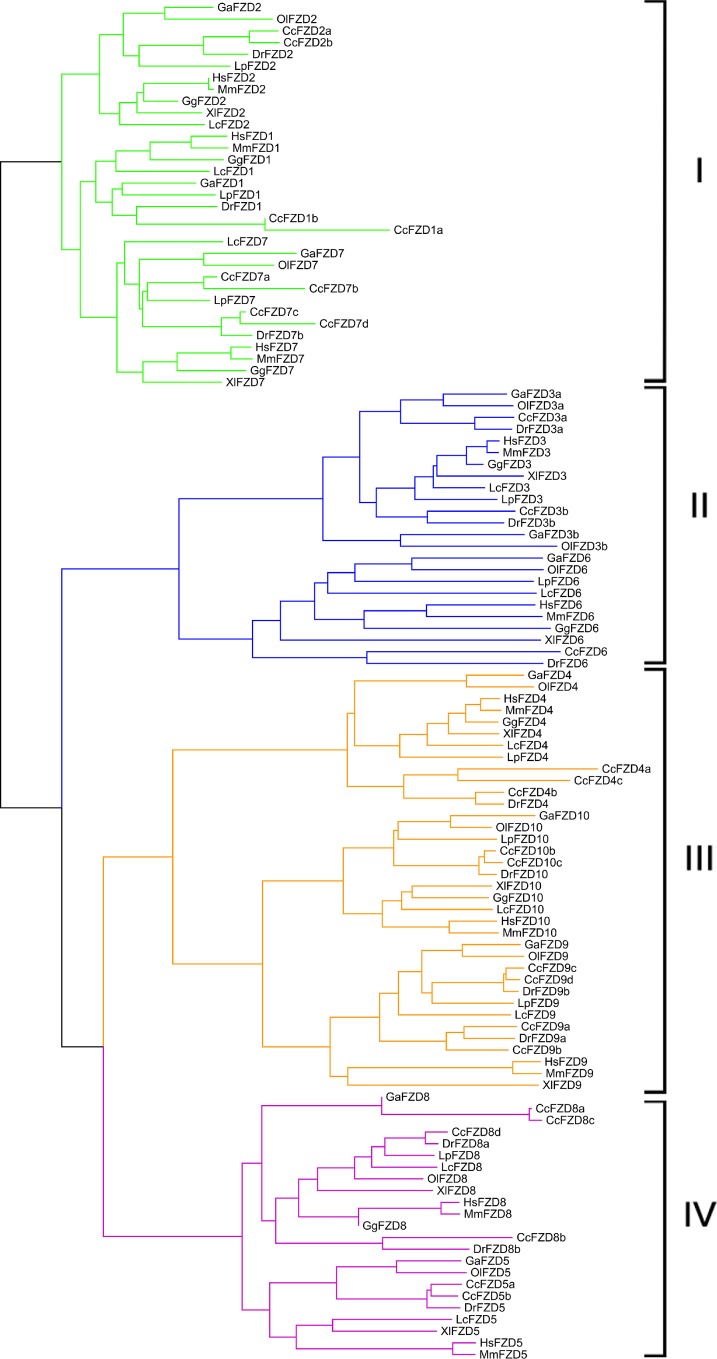
Neighbor-joining-based phylogenetic tree of 116 FZD protein sequences.

**Fig 2:**
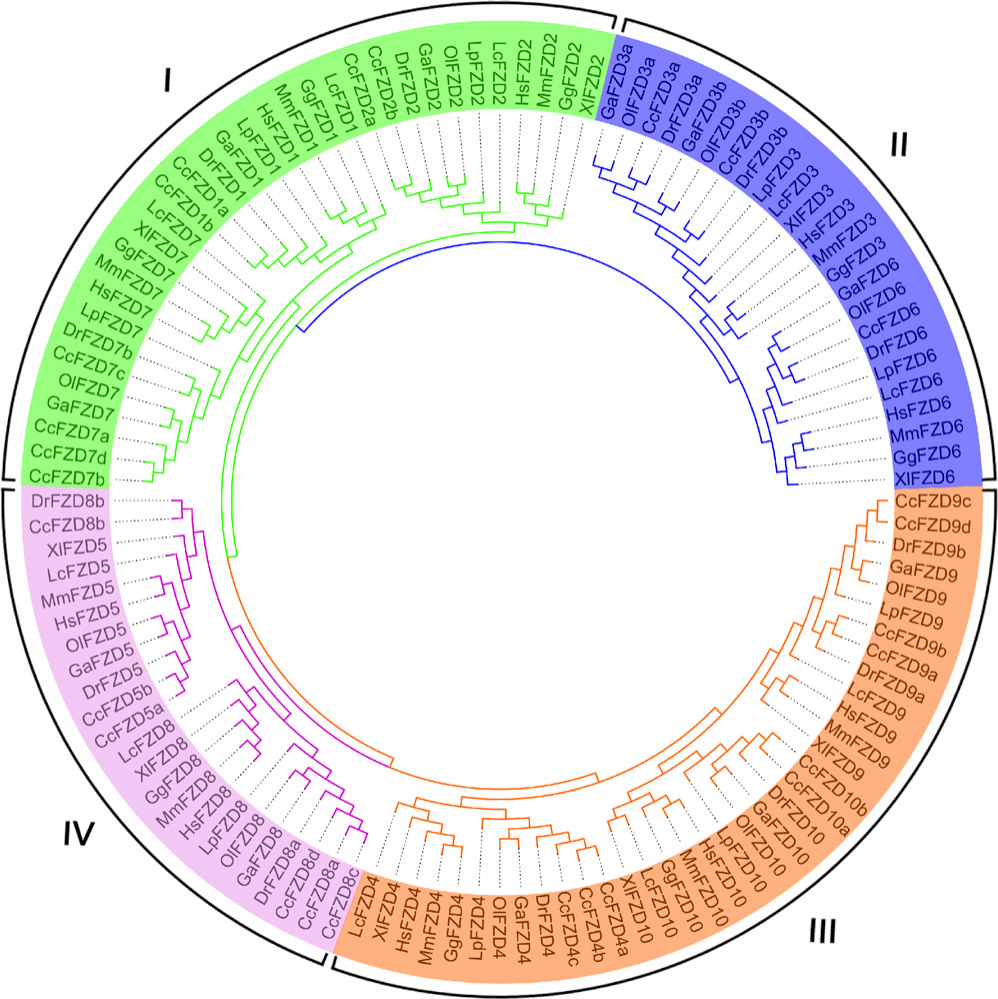
Maximum-likelihood-based phylogenetic tree of 116 FZD protein sequences.

To examine phylogenetic relationships of FZD genes in the teleosts and representative higher organisms, we collected a total of 116 FZD genes (S1 Table) from common carp (*Cyprinus carpio*, Cc), zebrafish (*Danio rerio*, Dr), medaka (*Oryzias latipes*, Ol), stickleback (*Gasterosteus aculeatus*, Ga), spotted gar (*Lepisosteus oculatus*, Lp), coelacanth (*Latimeria chalumnae*, Lc), African clawed frog (*Xenopus laevis*, Xl), chicken (*Gallus gallus*, Gg), mouse (*Mus musculus*, Mm), and human (*Homo sapiens*, Hs) for phylogenetic analysis. Two phylogenetic dendrograms constructed based on alignments of the amino acid sequences of the FZD proteins using both maximum likelihood (ML, Fig 1) and neighbor-joining (NJ, Fig 2) showed high topological consistency, indicating the reliability of the phylogenetic relationships of the FZD genes. The 116 vertebrate FZD genes formed four distinct polyphyletic groups corresponding to four subfamilies. The four subfamilies were characterized based on the topology: subfamily I, which contained *FZD1*, *FZD2*, and *FZD7*; subfamily II, which contained *FZD3* and *FZD6*; subfamily III, which contained *FZD4*, *FZD9*, and *FZD10*; and subfamily IV, which contained *FZD5* and *FZD8*. Based on these results, we inferred that all vertebrate FZD genes were likely derived from four ancestral FZD genes possessed by the common ancestor of cnidarians and bilaterians [45].

The 26 FZD genes identified from the common carp genome were clearly assigned into these four distinctive subfamilies. For gene annotation and nomenclature, the 26 FZD genes were first classified based on the topologies and their similarity to the 10 known zebrafish FZD orthologous genes; duplicated genes were assigned an alphabetic suffix (a, b, c, etc.). The nomenclature of the 26 common carp FZD genes is listed in Tables 1 and 2. We identified four copies of three FZD genes (*FZD7*, *FZD8*, and *FZD9*), three copies of one FZD gene (*FZD4*), two copies of five FZD genes (*FZD1*, *FZD2*, *FZD3*, *FZD5*, and *FZD10*), and one FZD gene (*FZD6*) with only one copy. Clearly, intensive gene duplication and expansion of the FZD gene must have occurred in common carp. The complexity of copy number and gene expansion suggest multiple gene or genome duplication events may have occurred in its evolution history.

**Table 2:** FZD gene family in the genomes of the ten vertebrates.

common carp	zebrafish	medaka	stickleback	spotted gar	coelacanth	clawed frog	chicken	mouse	human
26	14	10	11	9	10	10	8	10	10
FZD1a	FZD1	-	FZD1	FZD1	FZD1	FZD1	FZD1	FZD1	FZD1
FZD1b									
FZD2a	FZD2	FZD2	FZD2	FZD2	FZD2	FZD2	FZD2	FZD2	FZD2
FZD2b									
FZD3a	FZD3a	FZD3a	FZD3a	FZD3	FZD3	FZD3	FZD3	FZD3	FZD3
FZD3b	FZD3b	FZD3b	FZD3b						
FZD4a	FZD4	FZD4	FZD4	FZD4	FZD4	FZD4	-	FZD4	FZD4
FZD4b									
FZD4c									
FZD5a	FZD5	FZD5	FZD5	-	FZD5	FZD5	FZD5	FZD5	FZD5
FZD5b									
FZD6	FZD6	FZD6	FZD6	FZD6	FZD6	FZD6	FZD6	FZD6	FZD6
FZD7a	FZD7a	FZD7	FZD7	FZD7	FZD7	FZD7	FZD7	FZD7	FZD7
FZD7b	FZD7b								
FZD7c									
FZD7d									
FZD8a	FZD8a	FZD8	FZD8	FZD8	FZD8	FZD8	FZD8	FZD8	FZD8
FZD8b									
FZD8c	FZD8b								
FZD8d									
FZD9a	FZD9a	FZD9	FZD9	FZD9	FZD9	FZD9	-	FZD9	FZD9
FZD9b	FZD9b								
FZD9c									
FZD9d									
FZD10a	FZD10	FZD10	FZD10	FZD10	FZD10	FZD10	FZD10	FZD10	FZD10
FZD10b									

### Gene duplications and retention of FZDs

Often followed by some sort of divergence, gene duplications, played necessary roles in providing the raw material for the evolution of the species [46]. Duplicate genes were fixed and maintained within a population with 3 distinct outcomes: neofunctionalization, subfunctionalization, and conservation of function [47]. A number of models have been posed for the mechanisms responsible for duplicate gene retention; either because a slow rate of evolution correlated with other factors that predispose genes to be preserved in duplicate [48], or because the relative stoichiometry of proteins that belong to the same complex or to the same metabolic pathway must be maintained after the duplication [49]. It has been noted previously that transcription factors and genes involved in signal transduction are over-retained in duplicate after WGD, but not after smaller scale DNA duplication events [50, 51].

Based on molecular clocks, the 4RWGD in common carp was inferred to be around 8.2 million years ago [30]. Therefore, the significant expansion of FZD genes in the common carp genome may be the result of this additional WGD, which could have caused a sudden doubling of the FZD genes. As shown in Table 2, common carp retained double or more than double the FZD copies of the zebrafish FZD genes, except *FZD3* and *FZD6*, which strongly suggests that the 4RWGD event was the major contributor to FZD gene family expansion in common carp. Similar results were observed when to the common carp FZD genes were compared with the FZD genes in other teleost genomes. This result also suggests that gene loss was an infrequent occurrence in the tetraploid common carp genome because it has retained two almost complete sets of diploid genomes from its two ancient parent species.

**Fig 3:**
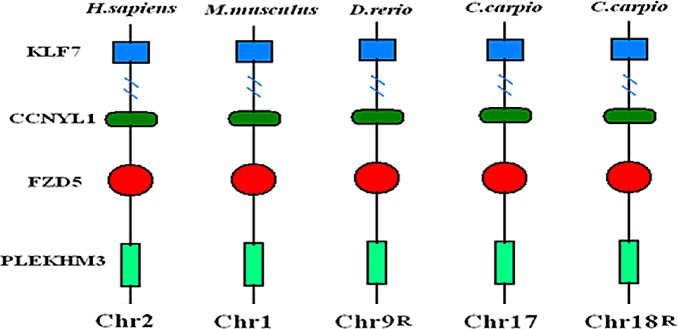
Schematic diagram showing the location of FZD5 and neighboring genes in syntenic regions of human, mouse, zebrafish, and common carp chromosomes.

Although mammals and teleosts last shared a common ancestor many hundred million years ago, a growing number of studies have reported extensive conserved synteny between the chromosomes of teleosts and mammals, which favors the rule of additional genome duplication in fishes [52]. We performed a syntenic analysis of *FZD5* across the common carp, zebrafish, human, and mouse genomes to confirm the 4RWGD. In common carp, the two copies of *FZD5* were distributed on two chromosomes, while zebrafish, human, and mouse each had only one copy. In all four genomes, the orthologous copies were harbored in conserved syntenic regions with a consistent gene order of *KLF7*, *CCNYL1*, *FZD5*, and *PLEKHM3* (Fig 3).

**Fig 4:**
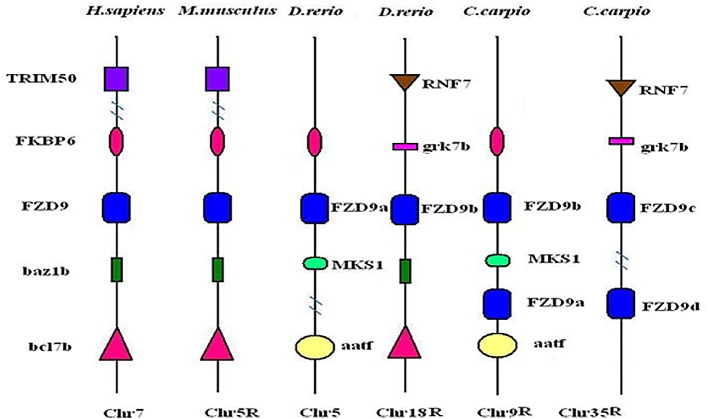
Schematic diagram showing the location of FZD9 and neighboring genes in syntenic regions of human, mouse, zebrafish and common carp chromosomes.

We also propose a special duplication style in the common carp genome. For example, in zebrafish, the two *FZD9* genes are on chromosomes 5 and 18, and, in common carp, the four *FZD9* genes were distribute on chromosomes 9 and 35 (Fig 4). Different evolutionary hypotheses have been suggested to explain the duplication an ancestral *FZD9* gene (named *FZD9*) in genome duplication events in teleosts. Here, we consider a new evolutionary scenario. Assuming that the putative teleost ancestor had the *FZD9* a/b genes on two different chromosomes (5 and 18), then, when genome duplication was finished in common carp, the four orthologs (*FZD9* a/b/c/d) of the zebrafish genes (*FZD9* a/b) were distributed on four different chromosomes (9, 10, 35, and 36). However, with the cis and trans mechanisms, in the common carp genome, the *FZD9* gene on chromosome 10 moved to chromosome 9 and the *FZD9* gene in chromosome 36 moved to chromosome 35, resulting in two FZD genes on each of the chromosomes (Fig 4). This hypothesis can explain the distribution of *FZD9* in the common carp genome reasonably.

We also observed duplications of the *FZD3*, *FZD7*, and *FZD8* genes in the zebrafish genome that likely resulted from the 3RWGD event. Compared with other teleost genomes, the zebrafish genome did not undergo complete diploidization after the WGD event and still retained duplicated FZD genes for the reasons discussed above. We identified four copies of *FZD7*, *FZD8*, and *FZD9* genes in common carp genome that likely derived from the two rounds of WGD without any gene loss during the evolution history. Although FZD gene expansion was derived largely from the 4RWGD event in common carp, several potential segmental duplications were also observed in the expanded FZD gene family. For instance, we found only one *FZD4* gene in each of the surveyed diploid teleost genomes (Table 2), but we identified three copies rather than two copies of *FZD4* in the common carp genome. The additional copy of *FZD4* could be derived from a segmental duplication, although the possibility that the diploid teleosts originally had two copies and the common carp four copies of *FZD4* cannot be excluded. In such a situation, gene loss events that removed one *FZD4* copy may have occurred in these genomes.

### Independent FZD gene loss in the teleosts

After duplication, one of the two redundant copies of a gene should theoretically be free to degenerate and become lost from the genome without consequence. Most gene pairs formed by a WGD have only a brief lifespan before one copy becomes deleted, leaving the other to survive as a single-copy locus. We observed that all the teleosts that experienced the 3RWGD retained two *FZD3* genes in their genomes; thus, theoretically, common carp should have retained four *FZD3* genes after the 4RWGD. Similarly, common carp had only one *FZD6* gene, which is universal in all other vertebrates. Under selection pressure, gene loss may have occurred quickly after the WGD events so as to maintain the single copy of *FZD6*. The *FZD6* protein sequences were found to be highly conserved across all the vertebrate species, suggesting that the conserved *FZD6* gene is critical for survivability and very little change is allowed in its coding sequence and copy number.

Although most teleosts have experienced the 3RWGD, we did not observe significant gene duplication and expansion of the FZD gene family in most of the studied teleost species (Table 2). Most of the FZD genes in the teleost genomes were single copies, just as they were in the genomes of higher organisms. This provides clear evidence of the re-diploidization that occurred after the WGD event for most of the genes and gene families. The functions of most genes are redundant after gene and genome duplications, and a single copy of a gene is usually sufficient to perform its function. Abundant copies of a single gene might accumulate detrimental mutations due to relaxed selection on one of the duplicates, which might gradually become a pseudogene and ultimately be lost in the genome. The process of re-diploidization could happen independently and progressively, as seems to have been the case in the zebrafish genome. Although the zebrafish genome still retains significant portions of duplicated genes (e.g., two copies of *FZD3*, *FZD7*, *FZD8*, and *FZD9*) and expanded gene families [53, 54], it is considered a diploid genome according to cytogenetic evidence. In the common carp genome, we could reasonably infer that the tetraploidization of the genome is gradual undergoing re-diploidization after 4RWGD; however, this is likely to be a long evolutionary process.

### Expression Profiling of FZD genes of common carp and Potentially Functional Inferences

FZDs have diverse roles in regulating cell proliferation and differentiation, and play essential roles in the earliest stages of embryonic development, during organogenesis. To determine how the spatial-temporal expression patterns of the closely-related members of the FZD gene family differ in common carp, we performed a RT-PCR analysis using gene-specific primers to check their expression levels in seven adult common carp tissues (brain, heart, spleen, liver, kidney, skin, and blood) and at different development stages post-fertilization (0 hours, 12 hours, 24 hours, 36 hours, 48 hours, 3 days, 5 days, and 7 days).

**Fig 5:**
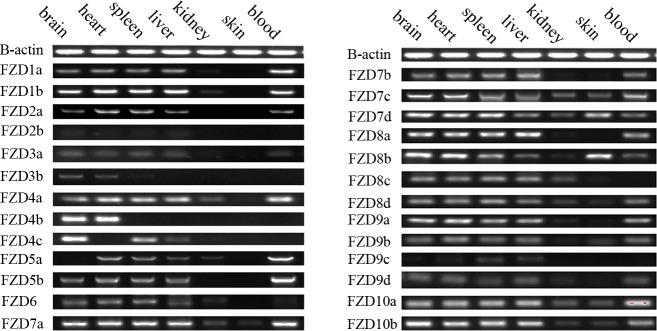
RT-PCR expression analysis of FZD genes in seven adult common carp tissues (brain, heart, spleen, liver, kidney, skin, and blood). The amplification of β-actin was used as an internal control. Gene names are indicated on the left of the panel.

As shown in Fig 5, the FZD gene family exhibited unique tissue-specific expression. In general, most of the FZD genes were widely expressed, but has a relatively high expression levels in brain, heart, spleen, liver and blood (Fig 5) and relative low expression levels in kidney and skin. Also, we observed that 23 of the FZD genes were widely expressed in four tissues (brain, heart, spleen, and liver), implying their important roles in organ development. *FZD7*d and *FZD8*b were highly expressed in skin, suggesting their specific expression and special functions in the development of skin in common carp.

Additionally, we observed significant expression differences in the duplicated FZD genes, providing evidence for gene subfunctionalization after WGD events. For instance, we found significant expression differences between *FZD2*a and *FZD2b*; namely, *FZD2*a was highly expressed in brain, heart, spleen, liver, and blood, while for *FZD2*b no expression was detected in any of the tissues tested. Similarly, we observed expression differences in the duplicated *FZD4*, *FZD5*, and *FZD9* genes. The functional divergence of duplicated genes may avoid potential adaptive conflicts and may have an important influence on the evolution of species.

**Fig 6:**
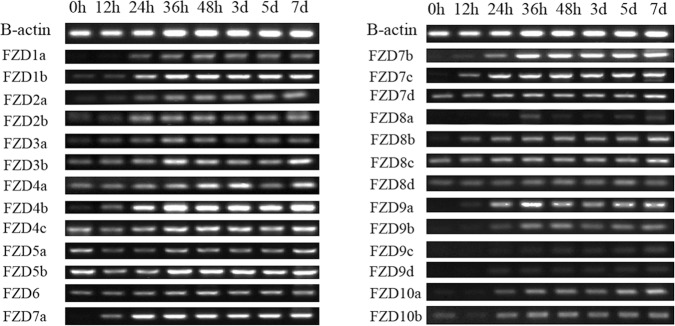
RT-PCR expression analysis of FZD genes in different development stages post-fertilization (0 hours, 12 hours, 24 hours, 36 hours, 48 hours, 3 days, 5 days, and 7 days). The amplification of β-actin was used as an internal control. Gene names are indicated on the left of the panel.

Gene expression levels in the different periods after fertilization are shown in Fig 6. In general, almost all of the FZD genes were widely expressed in all the time periods 24 hours post-fertilization, which indicated that most FZD genes played important role in the development stages after 24 hours. The exceptions were *FZD8*a, *FZD9*c, and *FZD9*d, which kept low expression levels in all the development stages. We also observed that only seven of the 26 genes (*FZD3*b, *FZD4*a, *FZD4*c, *FZD5*b, *FZD6*, *FZD7*d, and *FZD8*c) maintained relatively high expression levels in all the stages, which suggested they are important not only for the early stages of embryonic development, but also for development and physiological functions post hatch in common carp. Further, *FZD4*c, *FZD5*a, *FZD5*b, and *FZD10*b had high expression levels at the 0-hour than 12-hour stages, which indicated that these four genes were essential in the earliest embryonic development stages. To the contrary, *FZD3*a, *FZD4*b, *FZD7*a, *FZD7*c, and *FZD8*b showed no expression at the 0-hour stage but had relatively high expression at the 12-hour stage, which indicated that these genes were required for later embryonic development instead of the early stages.

Furthermore, almost all the duplicated FZD genes had the same significant expression at the different development stages, except for *FZD7*, *FZD8*, and *FZD9*. For instance, we found significant expression differences between *FZD7*a, *FZD7*b, *FZD7*c, and *FZD7*d; i.e., *FZD7*a and *FZD7*c were highly expressed 12 hours post-fertilization, while *FZD7*b was highly expressed after 24 hours, and *FZD7*d was highly expressed from 0 hours. Similarly, *FZD8*c and *FZD8*d had same gene expression patterns, while *FZD8*a and *FZD8*b each had different gene expression patterns. For *FZD9*, *FZD9*a and *FZD9*b expressed after the 24-hour stage and had the same expression patterns, while *FZD9*c and *FZD9*d had almost no gene expression at any of the development stages. The expression differences suggested that some of duplicated FZD genes only performed partial functions of their ancestral FZD genes, which are consistent with previous study based on large set of transcriptome.

## Materials and Methods

### Database analysis and sequence extraction

FZD genes from the nine surveyed species (human, mouse, chicken, clawed frog, spotted gar, medaka, stickleback, coelacanth, and zebrafish) were extracted from the Ensemble (http://asia.ensembl.org) genome databases. The genomes of these nine species have been well-characterized and annotated previously. Then, the 14 zebrafish FZD genes were used as query sequences to extract common carp FZD orthologous genes using BLAST tools. To collect all possible FZD copies in common carp, we also searched transcriptome datasets for FZD transcripts. Redundant gene sequences were removed after comparing the identified genome and transcriptome sequences.

### Gene characterization

To characterize the gene structure, we performed an exon-intron structure analysis using the Fancy Gene 1.4 online analysis tool (http://bio.ieo.eu/fancygene/). Then, to compare them with orthologous sequences in other genomes, we downloaded the annotated human and zebrafish FZD genes from the corresponding Ensemble genome databases. The structural components of the FZD genes were then compared across the three species. The gene structure of each surveyed common carp FZD genes was drawn in Fancy Gene 1.4.

### Phylogenetic analysis

To investigate the phylogenetic relationship with closely related teleost and model species, the FZD protein sequences of nine surveyed species were downloaded from the Ensemble databases. Then, the translated protein sequences of the common carp FZD orthologous genes and the FZD protein sequences from the nine other species (a total of 116 sequences) were aligned using ClustalW2 (http://www.ebi.ac.uk/Tools/msa/clustalw2/). The sequences were then manually trimmed of all sites that were not unambiguously aligned. The phylogenetic relationships of the 116 protein sequences were estimated using the ML and NJ approaches in the MEGA6 program [55]. Bayesian Information criterion values were calculated for each model to determine the optimum parameters. The Jones-Taylor-Thornton (JTT) + G and maximum composite likelihood models were used in the ML and NJ analyses, respectively. A total of 1000 bootstrap replicates were conducted for each calculation. The phylogenetic topologies were then visualized in Figtree 1.4.2 software (http://tree.bio.ed.ac.uk/software/figtree/).

### Gene nomenclature

The FZD orthologous genes in common carp were named based on their phylogenetic topologies, as well as their most related zebrafish genes. First, the subfamilies and gene members were determined for each common carp FZD orthologs based on the phylogenetic clades (for instance, *FZD1*, *FZD2*, etc.). Then, the closely related zebrafish FZD genes were assigned to each common carp FZD ortholog and the FZD genes were named after their most closely related zebrafish gene. When more than one copy of a common carp FZD gene was clustered with a certain zebrafish FZD gene, alphabetical suffixes were added to each copy (for instance, *FZD8*a, *FZD8*b, *FZD8*c, etc.). The names of each FZD gene in the surveyed species are listed in Tables 1 and 2.

### Expression profiling of FZD genes

Total RNAs were extracted from seven adult common carp tissues (brain, heart, spleen, liver, kidney, skin, and blood) and eight development stages post-fertilization (0 hours, 12 hours, 24 hours, 36 hours, 48 hours, 3 days, 5 days, and 7 days) using Trizol reagent (Life Technologies, Grand Island, NY, USA). The cDNA was synthesized by RT-PCR using a SuperScript III Synthesis System (Life Technologies). The β-actin gene was used as an internal positive control, with a forward primer (5′-TGCAAAGCCGGATTCGCTGG-3′) and a reverse primer (5′-AGTTGGTGACAATACCGTGC-3′). The PCR program comprised an initial denaturation step (4 min at 94°C), followed by 35 cycles of denaturation (30 s at 94°C), annealing (30 s at 56°C) and extension (1.5min at 72°C), and a final elongation step (7 min at 72°C). The PCR products were separated by gel electrophoresis (1.0% agarose gel at 140 V) in the presence of ethidium bromide and visualized under ultraviolet light.

### Syntenic analysis

Syntenic analyses were performed on selected FZD genes across the human, mouse, zebrafish, and common carp chromosomes by identifying the positions of FZD neighboring genes. The organization of the genes on the chromosomes of the model species was obtained from the Ensemble databases, while the gene organization of common carp was based on the draft sequences of the common carp genome assembly. Syntenic maps were then drawn based on the gene locations in the surveyed species.

## Conclusions

FZD genes play important roles in various biological processes, especially in critical signaling pathways for cell proliferation and embryo developing. In this study, we surveyed the common carp draft genome and identified a total of the 26 FZD copies, revealing the significant gene expansion of the FZD gene family in this species. Phylogenetic analysis of the FZD protein sequences performed across 10 species suggested that the FZD gene expansion found in the common carp genome was caused by WGD as well as segmental duplication events. Gene losses of FZD genes after the 4RWGD were also detected, which suggested the re-diploidization of certain duplicated genes or gene families after the WGD event. Through the expression profiling of FZD genes in common carp, we speculate that the ancestral gene was likely capable of performing all functions and was expressed broadly, while some descendant duplicate genes only performed partial functions and were specifically expressed at certain stages of development. Our results provide useful evidence for better understanding the FZD gene family of common carp, as well as gene fates and evolution after WGD events.

## Supporting Information

S1 Fig Exon–intron organization of common carp FZD genes. (TIF)

S1 Table The Ensemble accession numbers of nine species used in this manuscript. (XLS)
