# Supplementary figures and images for: Phylogenetic and Evolutionary Analyses of the Frizzled Gene Family in Common Carp (Cyprinus carpio) Provide Insights into Gene Expansion from Whole-Genome Duplications
Source: PLoS One. 2015 Dec 16;10(12):e0144037. doi: 10.1371/journal.pone.0144037 (PMC4686014; doi:10.1371/journal.pone.0144037)

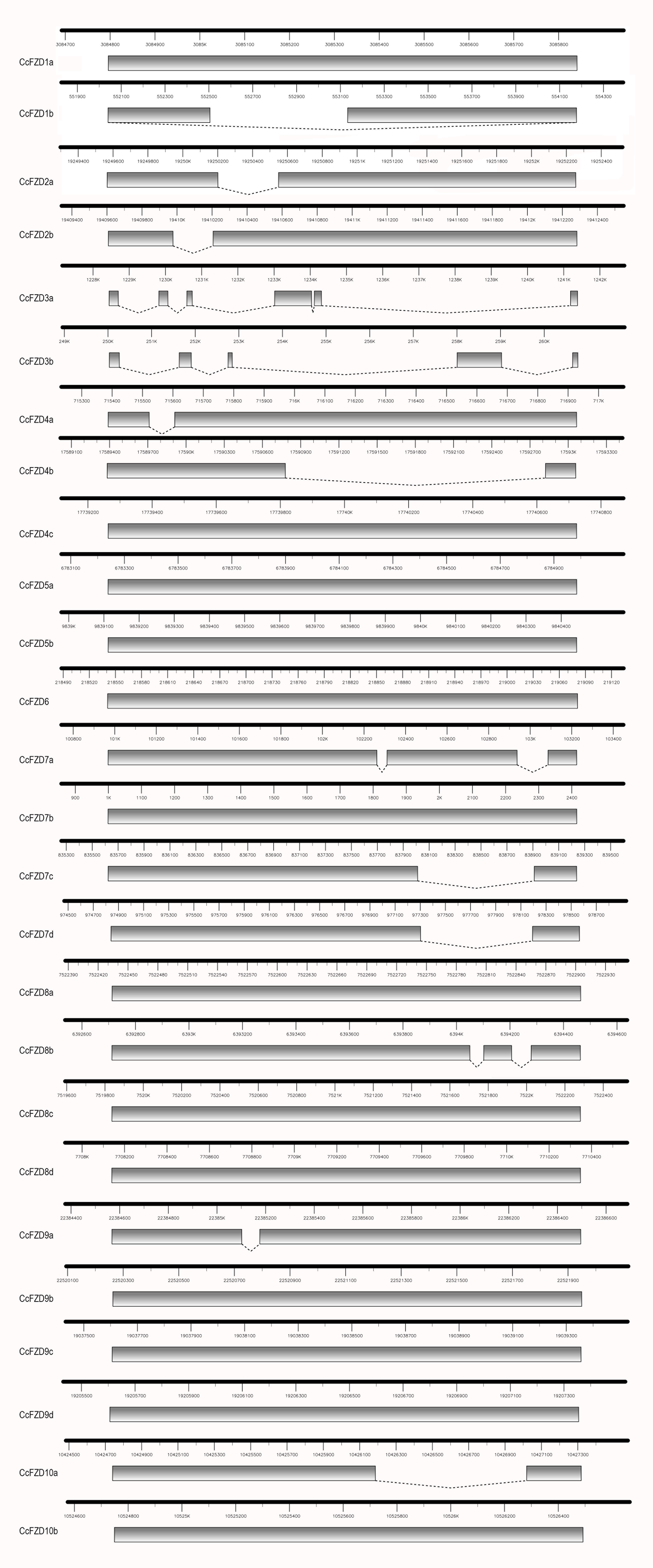

Supplement: S1 Fig — (TIF) [file pone.0144037.s001.tif]
